# Treatment outcomes of paul versus ahmed glaucoma implants

**DOI:** 10.1371/journal.pone.0338317

**Published:** 2025-12-23

**Authors:** Julia Prinz, Kira Hilmers, Constance Liegl, Peter Walter, Karl Mercieca, Verena Prokosch

**Affiliations:** 1 Department of Ophthalmology, Faculty of Medicine and University Hospital of Cologne, Cologne, Germany; 2 Department of Ophthalmology, RWTH Aachen University, Aachen, Germany; 3 Department of Ophthalmology, University of Bonn, Bonn, Germany; The University of Iowa, UNITED STATES OF AMERICA

## Abstract

**Objectives:**

To compare the outcomes of Paul (PGI) and Ahmed glaucoma implants (AGI) in patients with complex glaucoma.

**Methods:**

64 patients undergoing PGI and 40 patients undergoing AGI were included in this study. Intraocular pressure (IOP), the number of IOP-lowering eye drops, and complications were evaluated during an 18-month follow-up.

**Results:**

At 18 months, follow-up was completed by 26 patients (65.0%) in the AGI group and 45 patients (70.3%) in the PGI group. IOP was significantly reduced 18 months following PGI (12.3 ± 4.0 vs. 28.0 ± 9.3 mmHg, p < 0.001) and AGI (15.6 ± 5.2 vs. 30.7 ± 8.9 mmHg, p < 0.001) compared to preoperatively. The PGI achieved significantly lower IOP compared to the AGI group (p = 0.042). Similarly, the use of IOP-lowering eye drops decreased significantly at 18 months in the PGI (0.5 ± 0.8, p < 0.001) and AGI (1.3 ± 1.0, p < 0.001) groups, from baseline values of 3.3 ± 1.3 in PGI and 3.5 ± 1.3 in AGI. The number of IOP-lowering eye drops was significantly lower in the PGI than in the AGI group at both 12 (p = 0.031) and 18 months (p = 0.018). At the 18-month follow-up, qualified success rates for target pressures ≤18 mmHg were higher after PGI than AGI (IOP ≤ 18 mmHg: 66.6% vs. 84.2%, p = 0.017, ≤ 15 mmHg: 46.3% vs. 64.8%, p = 0.049, ≤ 12 mmHg: 24.9% vs. 43.0%, p = 0.047). There was no significant difference in the complication rates between PGI and AGI.

**Conclusion:**

Both PGI and AGI effectively reduced IOP and the number of IOP-lowering eye drops over an 18-month follow-up period. The PGI demonstrated significantly greater reductions in IOP and IOP-lowering eye drops than AGI at 18 months. The safety profiles of PGI and AGI were comparable.

## Introduction

Glaucoma is a group of diseases leading to progressive retinal ganglion cell (RGC) loss, ultimately resulting in irreversible blindness [1,2]. The number of people aged 40–80 years with glaucoma worldwide was estimated 76 million in 2020 and is expected to increase to 112 million by 2040 due to an aging population [3,4]. Elevated intraocular pressure (IOP) is the main risk factor for glaucoma and the mainstay of treatment [5].

Glaucoma drainage devices (GDD) are a treatment option in refractory glaucoma, but also a first-line therapy in patients with advanced glaucoma, conjunctival scarring, anterior segment dysgenesis, or in neovascular, congenital, aphakic, or uveitic glaucoma [6–10]. In patients with prior trabeculectomy, the multicenter randomized *Tube versus Trabeculectomy* (TVT) study showed a higher success rate and lower occurrence of early postoperative complications with GDD surgery compared to repeat trabeculectomy at a mean follow-up of 5 years [11].

GDDs divert aqueous humour from the anterior chamber to a subconjunctival base plate located in the equatorial region [12]. Different GDDs differ in size, end plate material composition, and whether they have a valve that restricts aqueous flow if IOP becomes too low [13]. To date, only a limited number of studies have directly compared existing GDDs [14]. The Ahmed Baerveldt Comparison (ABC) and Ahmed Versus Baerveldt (AVB) studies compared the Ahmed glaucoma implant, which has a valve (AGI, New World Medical, Rancho Cucamonga, California, USA) to the valveless Baerveldt glaucoma implant (BGI, Abbott Medical Optics, Abbott Park, Illinois) [13,15,16]. Both studies demonstrated that IOP was slightly [17] to significantly [13] lower following BGI compared to AGI. Additionally, a higher proportion of surgical failures was observed in the AGI group compared to the BGI group [17,18]. However, BGI was associated with a greater incidence of postoperative complications, including hypotony [13].

The Paul® glaucoma implant (PGI; Advanced Ophthalmic Innovations, Singapore, Republic of Singapore) is a novel valveless GDD designed to lower IOP in patients with refractory or complex glaucoma by providing effective aqueous humour drainage via a biocompatible and flexible silicone plate [19]. The internal (0.127 mm) and external (0.467 mm) tube diameters are significantly smaller compared to the AGI and BGI. The PGI’s base plate features a larger surface area (~342 mm²) compared to the AGI (~184 mm²) and is similar in size to the BGI (~350 mm²), facilitating enhanced aqueous drainage and potentially improving IOP control [20].

To date, only a few recent studies have compared the PGI to other GDDs, including the BGI [21], the AGI [22], and in a specific subgroup of silicone oil–related glaucoma [23]. Further evidence is needed to assess the relative efficacy and safety of the PGI in broader clinical settings. This study aimed to evaluate and compare the 18-month treatment outcomes and safety profiles of the non-valved PGI and the valved AGI.

## Materials and methods

### Patient characteristics

This retrospective study included 104 consecutive patients who underwent implantation of either the AGI (n = 40) or the PGI (n = 64) between October 2020 and January 2023. Only patients aged 18 years or older were included. Medical records were accessed for research purposes between September and October 2024 (15/09/2024–07/10/2024), after completion of the 18-month follow-up for all patients who underwent AGI or PGI surgery between October 2020 and January 2023. All data were anonymized prior to analysis. Clinical examinations were conducted preoperatively and at 1 day, 1 week, 6 weeks, 3, 6, 12, and 18 months postoperatively. All patients completed the preoperative examination as well as the postoperative assessments on day 1 and at week 1. In the AGI group, the 6-week, 3-, 6-, 12- and 18-month follow-up visits were completed by 36 (90.0%), 36 (90.0%), 35 (87.5%), 30 (75.0%), and 26 (65.0%) patients, respectively. In the PGI group, the 6-week, 3-, 6-, 12-, and 18-month follow-up visits were attended by 59 (92.2%), 56 (87.5%), 55 (85.9%), 55 (85.9%), and 45 (70.3%) patients, respectively. These examinations included IOP measurement, documentation of the number of IOP-lowering eye drops, assessment of complications, and evaluation of best-corrected visual acuity (BCVA). Additionally, demographic data, including age, sex, and ocular history, were recorded.

All surgeries were performed by two experienced surgeons (V.P. and K.M.) at the Department of Ophthalmology, University Hospital of Cologne and at the University Eye Hospital Bonn, Germany. The decision to use either a PGI or an AGI was at the discretion of the surgeon and was not based on predefined clinical criteria or a formal protocol. The study adhered to the tenets of Helsinki. Ethical approval for the study was obtained from the medical ethics committee of the University of Cologne (21–1539). Written informed consent was obtained from all participants. Complete and qualified success rates were defined based on IOP thresholds of ≤21 mmHg, ≤ 18 mmHg, ≤ 15 mmHg, and ≤12 mmHg. Complete success was achieved when these IOP targets were met without the use of IOP-lowering eye drops, while qualified success was defined as achieving these targets with or without the use of IOP-lowering eye drops.

Baseline demographic and clinical characteristics, including age (p = 0.191), gender (p = 0.547), and glaucoma subtypes, were evenly distributed between the AGI and PGI groups (Table 1). The number of prior ocular surgeries for all patients is detailed in Table 2.

**Table 1:** Patient characteristics in the Ahmed^®^ glaucoma implant (AGI) and Paul^®^ glaucoma implant (PGI) groups. POAG: primary open angle glaucoma, PSXG: pseudoexfoliative glaucoma.

	AGI	PGI	p-value
**n**	40	64	
**Age [years]**	59.8 ± 17.9	64.1 ± 15.6	0.191
**Female**	21 (52.5%)	28 (43.8%)	0.547
**Glaucoma Severity** **(Hodapp–Parrish–Anderson)**			
** **Mild	4 (10.0%)	6 (9.4%)	1.0
** **Moderate	17 (42.5%)	30 (46.5%)	0.690
** **Advanced	19 (47.5%)	28 (43.8%)	0.840
***Glaucoma Subtype***			
** **POAG	23 (57.5%)	42 (65.6%)	0.403
** **PSXG	4 (10.0%)	7 (10.9%)	0.880
** **Uveitic Glaucoma	5 (12.5%)	5 (7.8%)	0.322
** **Neovascular Glaucoma	5 (12.5%)	7 (10.9%)	0.521
** **Congenital Glaucoma	3 (7.5%)	3 (4.7%)	0.673
***Patch***			
** **Fascia Lata	21 (52.5%)	28 (43.8%)	
** **Cornea	12 (30.0%)	36 (56.3%)	
** **Tutopatch	7 (17.5%)	0 (0.0%)	

**Table 2:** Number and percentages of prior ocular surgeries in the Ahmed^®^ glaucoma implant (AGI) and Paul^®^ glaucoma implant (PGI) group. SLT: selective laser trabeculoplasty, Kahook^®^: Kahook Dual Blade (New World Medical, Rancho Cucamonga, CA, USA), XEN^®^: XEN gel stent (Allergan Inc., CA, USA), iStent^®^: iStent trabecular micro-bypass stent (Glaukos Corporation, Laguna Hills, CA, USA).

Ocular Surgery History	AGI	PGI	p-value
Phacoemulsification	35 (87.5%)	54 (84.4%)	0.445
Trabeculectomy	15 (37.5%)	32 (50.0%)	0.148
Trabeculectomy Revision	9 (22.5%)	14 (21.9%)	0.562
Vitrectomy	17 (42.5%)	18 (28.1%)	0.142
Cyclophotocoagulation	13 (32.5%)	16 (25.0%)	0.271
SLT	5 (12.5%)	7 (10.9%)	0.521
Canaloplasty	5 (12.5%)	5 (7.8%)	0.322
Kahook^®^ Goniotomy	5 (12.5%)	7 (10.9%)	0.521
Trabeculotomy	2 (5.0%)	3 (4.7%)	0.641
XEN^®^ Stent	2 (5.0%)	5 (7.8%)	0.450
Keratoplasty	3 (7.5%)	5 (7.8%)	0.633
iStent^®^	0 (0.0%)	3 (4.7%)	0.229

### Surgical technique

**Fig 1:**
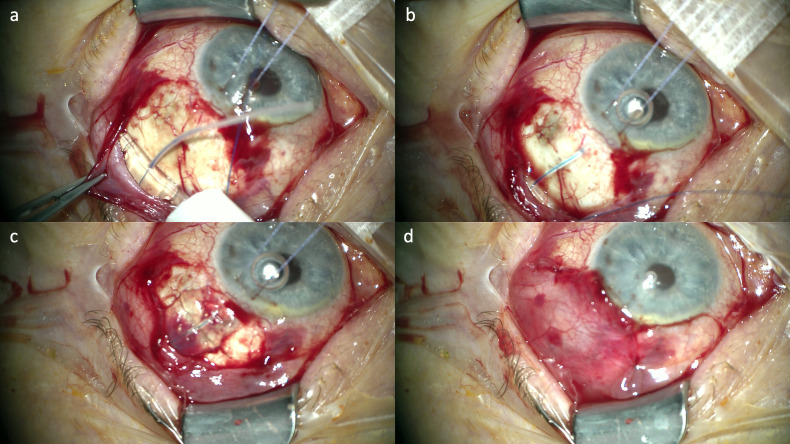
The base plate of a Paul® glaucoma implant is placed in the supero-temporal quadrant beneath the recti muscles. A 6−0 non-absorbable monofilament polypropylene suture (Prolene, Ethicon, Johnson & Johnson Medical GmbH, Norderstedt, Germany) is partly inserted into the tube to reduce immediate aqueous humour flow and to prevent early postoperative hypotony **(a)**. The tip of the tube is guided through a deep scleral tunnel into the anterior chamber **(b)**. The part of the tube that lies on the sclera is covered with a corneal donor patch **(c)**. The conjunctiva is closed using Vicryl 8−0 (Ethicon) **(d)**.

The procedure for AGI and PGI implantations has been reported previously [24,25]. All surgeries were performed in general or sub-Tenon’s anaesthesia. Following a fornix-based conjunctival incision, cautery was applied gently to the scleral bed. Mitomycin C 0.5 mg/ml was applied to the subconjunctival space using three corneal shields for 3 minutes, followed by an irrigation with balanced salt solution (Alcon BSS, Alcon Pharma GmbH, Freiburg, Germany). Afterwards, the plate was placed 8−10 mm from the limbus and sutured to the sclera with 8−0 non-absorbable nylon (Ethicon) sutures. For PGI, one end of a 6−0 non-absorbable monofilament polypropylene suture (Prolene, Ethicon) was inserted into the tube to reduce immediate flow (Fig 1). Then, the tube was cut to ensure a short length within the anterior chamber. The intraluminal suture was pushed to the tip of the tube. A deep scleral tunnel was created with a bent 26 Gauge needle. The tip of the tube was inserted through the tunnel into the anterior chamber. Then, the tube was sutured to the sclera using 8−0 nylon sutures (Ethicon). For PGI, the distal end of the intraluminal suture was placed into an infero-temporal conjunctival pocket.

The part of the tube that lies on the sclera was patched with allogenous Tutoplast^®^ fascia lata (fascia lata, Bess Medizintechnik GmbH, Berlin, Germany) or anterior lamellae from allogenous corneas which were provided by the corneal bank of the Department of Ophthalmology, University of Cologne, Germany. The patch was sutured to the sclera with 8−0 nylon sutures (Ethicon). Finally, the conjunctiva was closed with Vicryl 8−0 (Ethicon).

Postoperatively, dexamethasone 1 mg/ml eye drops (Dexa-sine SE, Alcon Pharma GmbH, Freiburg, Germany) were applied 8 times daily for 1 week tapering by one drop a week thereafter. Additionally, patients were treated with ofloxacin 3.0 mg/ml (Floxal EDO, Dr. Gerhard Mann chem.-pharm. Fabrik GmbH, Berlin, Germany) eye drops 4 times daily for 1 week.

### Statistical analysis

Statistical analysis was conducted using GraphPad Prism (Version 9.0 for Windows). All values are presented as mean ± standard deviation. All continuous variables were tested for normality using the Shapiro–Wilk test. As the data were normally distributed, group differences were evaluated using independent-sample *t*-tests for continuous variables, while proportions for categorical variables were compared using the Chi-square test. A p-value of <0.05 was considered statistically significant. In addition, a post hoc power analysis (two-sample t-test, two-sided, α = 0.05) was conducted to evaluate whether the study was adequately powered to detect a clinically relevant difference in IOP reduction between the AGI and PGI groups.

## Results

**Fig 2:**
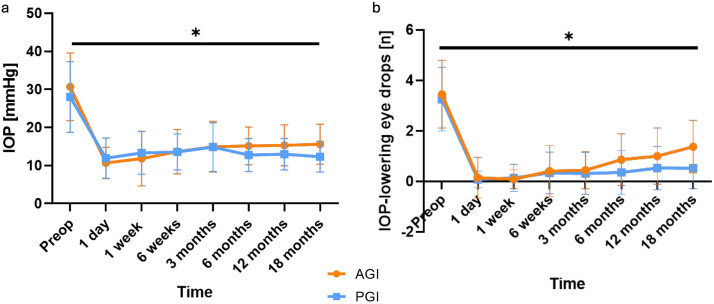
Mean intraocular pressure (IOP, a) and the mean number of IOP-lowering eye drops (b) preoperatively (preop) and at 1 day, 1 week, 6 weeks, and 3, 6, 12, and 18 months after Ahmed^®^ glaucoma implant (AGI) and PAUL^®^ glaucoma implant (PGI). Compared to preop, IOP was significantly (*) reduced at all follow-ups in both the AGI and PGI group (p < 0.001). At the 18-month follow-up, IOP was significantly lower following PGI than AGI (p = 0.042). Similarly, the number of IOP-lowering eye drops was significantly lower in the PGI compared to the AGI group at the 12- (p = 0.031) and 18-month follow-up (p = 0.018).

Post hoc power analysis revealed that with the available sample size (AGI n = 40, PGI n = 64), the study had > 90% power to detect a between-group difference of 3 mmHg in IOP reduction at 18 months. Preoperatively, IOP was comparable between the AGI (30.7 ± 8.9 mmHg) and PGI groups (28.0 ± 9.3 mmHg, p = 0.152, Fig 2). Compared to preoperatively, IOP was significantly reduced following AGI and PGI through 18 months of follow-up (all follow-ups: p < 0.001). At the 18-month follow-up, IOP was significantly lower after PGI compared to AGI (p = 0.042). The number of IOP-lowering eye drops was comparable preoperatively (AGI 3.5 ± 1.3, PGI 3.3 ± 1.3, p = 0.321) and was significantly reduced at all follow-ups in the AGI (p < 0.001) and PGI group (p < 0.001, Fig 2). At the 12- (p = 0.031) and 18-month follow-up (p = 0.018), the number of IOP-lowering eye drops was significantly lower after PGI compared to AGI.

Best corrected visual acuity (BCVA) was comparable preoperatively (AGI 0.8 ± 0.4 logMAR, PGI 0.7 ± 0.5, p = 0.128). At the first postoperative day, BCVA was significantly worse following AGI (0.9 ± 0.3 logMAR, p = 0.029) and PGI (0.7 ± 0.5 logMAR, p = 0.002). There were no significant changes in BCVA at any other follow-up in the AGI or PGI group compared to preoperatively (18 months AGI: p = 0.966; PGI: p = 0.875).

In the PGI group, the intraluminal prolene suture was removed in 28 patients (43.8%) after a mean postoperative time of 3.2 ± 2.6 months. This resulted in a mean reduction of IOP from 20.9 ± 4.1 mmHg to 12.5 ± 2.7 mmHg (−7.9 ± 3.9 mmHg).

**Fig 3:**
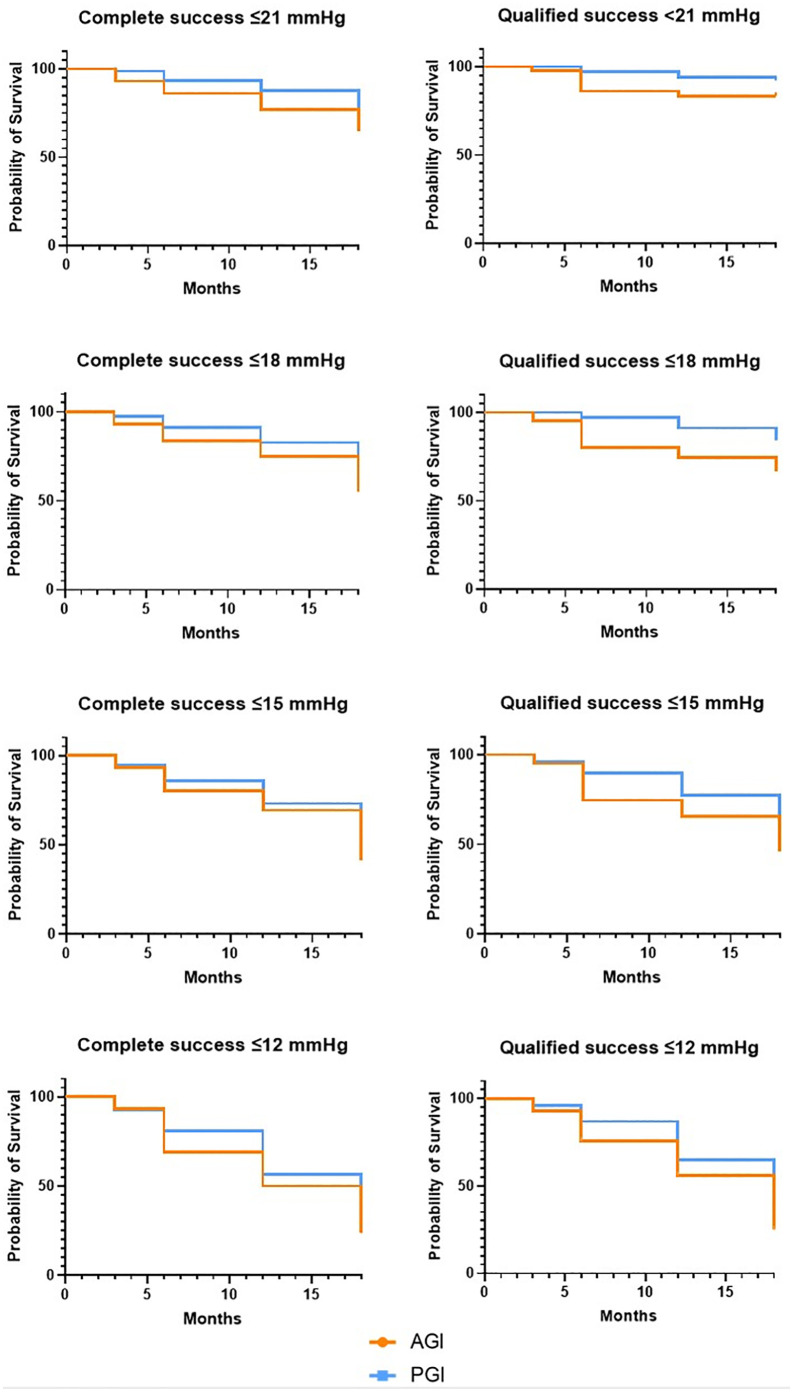
Kaplan-Meier curves for the Ahmed^®^ (AGI, orange) and PAUL^®^ glaucoma implant (PGI, blue) groups. Complete and qualified success rates for intraocular pressure ≤ 21, 18, 15, and 12 mmHg.

The Kaplan-Meier survival curves (Fig 3) illustrate the complete and qualified success rates for IOP thresholds of ≤21 mmHg, ≤ 18 mmHg, ≤ 15 mmHg, and ≤12 mmHg in the AGI and PGI groups. The complete success rates for IOP ≤ 21 mmHg were 76.3% in the PGI group and 65.0% in the AGI group (p = 0.150). The complete success rates were also comparable between AGI and PGI for IOP thresholds of ≤18 mmHg (66.5% vs. 55.1%, p = 0.211), ≤ 15 mmHg (52.7% vs. 41.6%, p = 0.945), and ≤12 mmHg (29.0% vs. 23.6%, p = 0.353).

Qualified success rates were comparable between AGI and PGI for IOP ≤ 21 mmHg (83.4% vs. PGI 92.1%, p = 0.112). Qualified success rates were significantly higher after PGI compared to AGI for IOP ≤ 18 mmHg (66.6% vs. 84.2%, p = 0.017), ≤ 15 mmHg (46.3% vs. 64.8%, p = 0.049), and ≤12 mmHg (24.9% vs. 43.0%, p = 0.047).

### Complications

The most common postoperative complication observed in this study was macular oedema, occurring in 16.7% of patients in the AGI group and 4.5% in the PGI group (p = 0.075, Table 3). In four of the five AGI cases of macular oedema, topical nepafenac was administered and led to resolution, while one patient received a single parabulbar injection of triamcinolone. Both PGI cases were treated with topical Nepafenac, resulting in complete resolution. According to the World Glaucoma Association guidelines, we distinguished between numerical hypotony and hypotony requiring intervention; in our cohort, all cases were transient, and none were associated with structural or functional sequelae necessitating treatment. Numerical hypotony, defined as an IOP < 6 mmHg at any postoperative time point, was the second most frequent complication, observed in 5.0% of patients in the AGI group and 3.1% in the PGI group (p = 1.0). Vitreous haemorrhage and anterior chamber haemorrhage were infrequent complications comparably distributed between the two groups, which resolved spontaneously without surgical intervention in all cases. Corneal decompensation occurred in one patient in the AGI group and two patients in the PGI group, all of whom had undergone prior keratoplasty. The drainage tubes were appropriately positioned in all cases, with no risk of corneal contact, so tube repositioning was not required. These cases were closely monitored, no patient required repeat keratoplasty during the follow-up period. Aqueous misdirection occurred in one patient per group within the first 3 postoperative months. The PGI case was successfully managed with cycloplegics and Nd:YAG laser hyaloidotomy. The AGI case required pars-plana vitrectomy with disruption of the anterior hyaloid to relieve the misdirection. Conjunctival erosion occurred in one patient in the AGI group and two patients in the PGI group. In all cases, the exposed drainage tube was surgically covered with a renewed corneal patch graft, resulting in complete resolution without the need for repeat surgery.

**Table 3:** Postoperative complications between Ahmed^®^ glaucoma implant (AGI) and Paul^®^ glaucoma implant (PGI) surgery.

Complication	AGI	PGI	p-value
Hypotony	2 (5.0%)	2 (3.1%)	1.0
Hypotony Requiring Intervention	0 (0.0%)	0 (0.0%)	–
Vitreous Haemorrhage	1 (3.3%)	0 (0.0%)	0.385
Anterior Chamber Haemorrhage	1 (3.3%)	2 (4.5%)	1.0
Macular Oedema	5 (16.7%)	2 (4.5%)	0.075
Corneal Decompensation	1 (3.3%)	2 (4.5%)	1.0
Aqueous Misdirection	1 (3.3%)	1 (2.3%)	1.0
Conjunctival Erosion	1 (3.3%)	2 (4.5%)	1.0

## Discussion

According to our main findings, IOP and IOP-lowering eye drops were significantly reduced through 18 months of follow-up both in the AGI and PGI group. The PGI exhibited superior outcomes in terms of IOP control and medication reduction compared to the AGI at 18 months, while maintaining a comparable safety profile. PGI was superior to AGI for target pressures below 18 mmHg regarding qualified success rates at the 18-month follow-up.

In patients undergoing AGI, IOP was significantly reduced from 30.7 ± 8.9 mmHg to 15.3 ± 5.4 mmHg after 12 months and to 15.6 ± 5.2 mmHg after 18 months. Similarly, in the *Ahmed versus Baerveldt (AVB)* study, IOP was reduced from 31.1 mmHg to 16.5 mmHg after 1 year and to 16.6 mmHg after 5 years in patients undergoing AGI [18]. The *Ahmed Baerveldt Comparison (ABC) study* reported a preoperative IOP of 31.2 mmHg in patients undergoing AGI, which was significantly reduced to 15.4 mmHg at the 12-month follow-up and to 14.7 mmHg at the 5-year follow-up [16]. Also, the reduction of IOP-lowering eye drops in our study from 3.5 ± 1.3 to 1.3 ± 1.0 after 18 months is consistent with the outcomes of the AVB study reporting a reduction of IOP-lowering eye drops from 3.1 ± 1.0 to 1.8 ± 1.5 after 12 months [15].

In our study, PGI led to a significant reduction of IOP from 28.0 ± 9.3 mmHg to 13.0 ± 4.2 mmHg after 12 months and to 12.3 ± 4.0 mmHg after 18 months. Our results are comparable to previous studies, reporting an IOP reduction from 34.3 mmHg to 13.2 mmHg after 12 months [19] and from 19.8 mmHg to 13.9 mmHg after 24 months [26]. IOP-lowering eye drops were reduced from 3.2 to 0.3 after 24 months in a previous study by Tan et al. [26] and from 3.3 to 0.5 after 18 months in our study.

Altogether, the IOP lowering efficacy of AGI and PGI in our study is largely consistent with previous studies. Up to the 3-month follow-up, IOP was higher in the PGI group than in the AGI group; this trend reversed thereafter. This observation can be most likely attributed to the intraluminal prolene suture, which was removed in 28 patients (43.8%) at a mean follow-up of 3 months postoperatively, resulting in a subsequent decrease in IOP. At the 18-month follow-up, IOP and IOP-lowering eye drops were significantly lower after PGI than after AGI, which may suggest long-term superiority of PGI over AGI in our patient cohort.

To date, the literature comparing PGI to AGI remains limited. Recently, the initial results of the multicentre randomized controlled trial *Paul Ahmed Comparison* (PAC) study were published, evaluating the efficacy and safety of AGI and PGI in paediatric patients with refractory glaucoma [27]. Patients were randomized to receive either the PGI or the AGI, with primary outcomes including IOP reduction and the number of IOP-lowering eye drops required at 12 months postoperatively. Secondary outcomes included the rates of surgical complications, need for additional interventions, and overall surgical success. The study revealed that both devices achieved significant reductions in IOP and the number of IOP-lowering medications at 12 months. While the PGI demonstrated a greater reduction in both parameters compared to the AGI, these differences were not statistically significant [27]. The success rate, defined as a postoperative IOP between 6 and 21 mmHg with or without IOP-lowering eye drops, was 80.0% in the PGI group and 73.6% in the AGI group. These findings closely align with the results of our study, where the qualified success rates for IOP ≤ 21 mmHg were similarly comparable between the AGI and PGI groups (83.4% vs. 92.1%, respectively).

The authors hypothesize that with a longer follow-up period, the superior outcomes of the PGI compared to the AGI observed in this study may become statistically significant. This hypothesis is supported by findings from a recent meta-analysis comparing the effectiveness of the non-valved Baerveldt Glaucoma Implant (BGI) to the AGI in childhood glaucoma [28]. The meta-analysis included 1,480 eyes from 32 studies and demonstrated a statistically significant higher success rate for the BGI group (87.8%) compared to the AGI group (86.6%) at one year postoperatively. The success rate differences became more pronounced over time, with the BGI group achieving significantly higher success rates at 2 years (82.5%) and 3 years (79.1%) compared to the AGI group, which declined to 62.8% and 43.2%, respectively, during the same periods.

Similarly, while the trend of greater success with the PGI at 12 months was not statistically significant in our study, this difference became significant at 18 months. This aligns with the findings of the BGI meta-analysis, which demonstrated that valveless GDDs may provide more sustainable long-term outcomes than valved devices [28]. These observations further support the notion that, particularly in the long term, the PGI may offer superior efficacy in managing refractory glaucoma.

Regarding safety, the PAC study reported comparable complication rates between the two groups, mainly including postoperative hypotony or tube erosion through the conjunctiva [27]. Postoperative hypotony occurred in 1 patient (4.0%) and in 2 patients (10.5%) in the AGI group. Despite being a valveless device, the PGI did not result in a higher incidence of postoperative hypotony compared to the AGI, neither in the initial results of the PAC study nor in our study. This is most likely attributable to the smaller lumen of the PGI’s drainage tube and the intraluminal prolene suture which further restricts aqueous humor flow in the early postoperative period [27].

In our study, macular oedema occurred more frequently following AGI implantation compared to PGI (16.7% vs. 4.5%), although this difference did not reach statistical significance. A likely contributing factor is the higher prevalence of comorbidities in the AGI group. Uveitic glaucoma accounted for 15.0% of AGI cases compared to only 6.3% in the PGI group, while secondary glaucoma due to proliferative diabetic retinopathy (PDR) was present in 10.0% of AGI cases versus 4.7% in the PGI group. Both uveitis and diabetic retinopathy are associated with blood-retinal barrier dysfunction, leading to increased vascular permeability and a heightened risk of postoperative cystoid macular oedema [29,30]. Additionally, intrinsic design differences may contribute to the higher incidence of macular oedema observed with the AGI. Hypothetically, the PGI’s smaller lumen and flow-restricting measures, such as the intraluminal suture, promote a more controlled IOP reduction, reducing mechanical stress to the retina and macular oedema through inflammatory responses.

Future studies with larger sample sizes and longer follow-up periods are warranted to further investigate these associations and to determine whether the AGI results in a higher incidence of macular oedema compared to the PGI.

Mendoza-Moreira et al. included 52 glaucoma patients in a comparative study evaluating the efficacy and safety of the PGI versus AGI [22]. Their findings demonstrated that PGI and AGI provided comparable IOP reduction with similar success rates [22]. However, their study was limited to a 12-month follow-up period, underscoring the need for longer-term data, as those presented here with an 18-month follow-up. These results add to the growing body of evidence supporting the PGI as a safe and effective alternative to the well-established AGI.

A retrospective study by Karapapak and Olgun evaluated the one-year outcomes of PGI versus the AGI in patients with secondary glaucoma due to silicone oil emulsification [23]. The study included 36 patients, with 18 receiving the PGI and 18 receiving the AGI. Both groups exhibited significant reductions in IOP and the number of IOP-lowering eye drops over the 12-month follow-up period. Specifically, the mean IOP decreased from 40.0 ± 13.0 mmHg preoperatively to 13.5 ± 2.2 mmHg postoperatively in the PGI group, and from 39.3 ± 10.0 mmHg to 14.9 ± 4.2 mmHg in the AGI group. The mean number of eye drops decreased from 3.8 ± 0.4 to 1.7 ± 1.3 in the PGI group, and from 4 to 1.9 ± 1.8 in the AGI group. Surgical success was achieved in 94% of eyes in the PGI group and 89% in the AGI group [23]. While both implants demonstrated similar efficacy, the PGI group experienced fewer complications requiring medical or surgical intervention. The authors concluded that longer-term studies with larger sample sizes are needed to establish the relative advantages of these procedures [23].

Our study has several limitations, including its retrospective design and the relatively short follow-up period of 18 months. Long-term studies with larger sample sizes are necessary to confirm the sustained efficacy and safety of the PGI compared to the AGI. Additionally, future research should explore patient-reported outcomes and quality of life measures to provide a more comprehensive assessment of these surgeries. Another limitation of our study is the inherent difficulty in comparing outcomes across studies due to the lack of standardized definitions of surgical success. Unlike some studies that include low IOP limits (>6 mmHg) or percentage reductions from baseline in their criteria, we defined success solely based on an upper IOP limit without considering low IOP values. This approach aligns with the European Glaucoma Society and World Glaucoma Association recommendations, which state that IOP measurements below 6 mmHg are may occur postoperatively and should be reported, but only clinically relevant hypotony should be classified as an adverse event [31,32]. Our definition may limit direct comparability with studies employing different success criteria, however, it avoids misclassification of transient or asymptomatic hypotony as failure.

## Conclusion

AGI and PGI resulted in a significant reduction of IOP and the number of IOP-lowering eye drops through 18 months of follow-up. IOP was significantly lower in the PGI compared to the AGI group at the 18-month follow-up. The number of IOP-lowering eye drops was significantly lower following PGI than AGI at the 12- and 18-month follow-up. PGI was superior to AGI for target pressures below 18 mmHg regarding qualified success rates at the 18-month follow-up.
